# A training, assessment and feedback package for the trainee shoulder sonographer

**DOI:** 10.1177/1742271X14566067

**Published:** 2015-01-28

**Authors:** Michael J Smith, Alison Rogers, Nazar Amso, Julia Kennedy, Alison Hall, Peter Mullaney

**Affiliations:** 1 School of Healthcare Sciences, Cardiff University, Cardiff, UK; 2 Institute for Translation, Innovation, Methodology and Engagement, Cardiff University School of Medicine, Cardiff, UK; 3 Primary Care Sciences, Keele University, Staffordshire, UK; 4 Radiology Department, University Hospital of Wales, Cardiff, UK

**Keywords:** Ultrasound, sonography, training, assessment, shoulder, musculoskeletal

## Abstract

Diagnostic ultrasound of the shoulder is recognised as being one of the most technically challenging aspects of musculoskeletal ultrasound to master. It has a steep learning curve and makes gaining competency a time-intensive training process for both the trainee and their trainer. This article describes a training, assessment and feedback package developed within the framework of a Consortium for the Accreditation of Sonographic Education approved post-graduate ultrasound course. The package comprises: (i) a shoulder diagnostic ultrasound scan protocol with definition of findings, differential diagnosis and pro forma for recording scan findings, (ii) an assessment form for performance of shoulder diagnostic ultrasound scans with assessment criteria and (iii) a combined performance assessment and scan findings form, for each tissue being imaged. The package has been developed using medical education principles and provides a mechanism for trainees to follow an internationally recognised protocol. Supplementary information includes the differential diagnostic process used by an expert practitioner, which can otherwise be difficult to elicit. The package supports the trainee with recording their findings quickly and consistently and helps the trainee and trainer to explicitly recognise the challenges of scanning different patients or pathologies. It provides a mechanism for trainers to quantify and trainees to evidence their emerging competency. The package detailed in this article is therefore proposed for use in shoulder ultrasound training and its principles could be adapted for other musculoskeletal regions or other ultrasound disciplines.

## Background

Diagnostic ultrasound of the musculoskeletal (MSK) system is a rapidly developing and in-demand technique which is increasingly recognised as a valuable diagnostic method, having several advantages over magnetic resonance imaging (MRI).^1^ Clinicians from a spectrum of professional backgrounds (including Radiology, Radiography, General Practice, Physiotherapy and Sports Medicine) are undertaking ultrasound training to enhance their clinical practice.^1–3^ However, diagnostic ultrasound is technically challenging and has a steep learning curve.^4–6^ Adapting technique to accommodate technical issues and patient presentation difficulties to generate consistent diagnostic images can be challenging as is the interpretation of subsequent images to arrive at a well-reasoned, differential diagnosis.^5^

As an unregulated imaging modality, diagnostic ultrasound can be used without formal training. However, the Consortium for the Accreditation of Sonographic Education (CASE)^7^-approved post-graduate courses are advocated by professional bodies such as the British Medical Ultrasound Society (BMUS)^8^ so that trainees can learn and evidence their competency in areas such as ultrasound physics, safety, anatomy and pathology-specific knowledge. However, gaining competency in effective scanning technique and differential diagnosis presents a significant challenge and this restricts the availability of training opportunities.

There is, therefore, a need to support both trainees and trainers in the gaining of competency in performing diagnostic ultrasound. This article describes a training, assessment and feedback package developed within the framework of a CASE approved post-graduate diagnostic ultrasound course.

## Diagnostic ultrasound of the shoulder

Diagnostic ultrasound can provide valuable clinical information to guide the management of soft tissue disorders of the shoulder.^4^ However, it is recognised as being particularly technically challenging^9^ due in part to the complex three-dimensional anatomy of the rotator cuff, technical issues specifically related to tendon ultrastructure and the co-operation required from the patient to visualise certain structures.

Various protocols for the sonographic assessment of the shoulder are available, and for the MSK trainee these can be supplemented by published articles, texts, online videos and short courses. However, it is in the clinical setting where the MSK trainee gains the required practical knowledge, experience and feedback. This includes observation of an expert practitioner’s technique performing the scan and the expert’s interpretation of the generated images, followed by the trainee generating images of an appropriate quality, managing technical or patient presentation difficulties and correctly interpreting the subsequent images. Time-wise, these are costly processes but are essential for the development of a competent sonographer.

## Training, assessment and feedback package

The training, assessment and feedback package described in this article comprises three main sections (see Appendix):

1. A shoulder diagnostic ultrasound scan protocol with definitions of findings, differential diagnosis and pro forma for recording scan findings.
2. An assessment form for performance of shoulder diagnostic ultrasound scans with assessment criteria.
3. A combined performance assessment and scan findings form, for each tissue being imaged.

## Section 1a: Shoulder diagnostic ultrasound scan protocol and Section 1b: Definition of findings and differential diagnosis

The importance of adhering to a scan protocol, particularly for a trainee, is widely accepted within sonography.^6,9^ It ensures that no aspects of a scan are omitted and that the scan is performed in a manner that is reproducible between scans and between sonographers. Numerous scan protocols for the shoulder are described in the literature and many sonographers will, over time, develop their own preferred way of performing a scan. As a European professional body comprising experts in the field of MSK sonography, the European Society of Musculoskeletal Radiology’s (ESSR) protocol for the shoulder^10^ was selected as the basis for the scan protocol used within this assessment and feedback package.

Whilst standardised approaches to performing a scan are available, the formulation of a differential diagnosis requires significant experience and detailed knowledge of relevant pathology. In shoulder sonography, areas of controversy or subjectivity include the appearance of tendinopathic changes and how these are differentiated from partial thickness supraspinatus tears.^11^ These areas of controversy or subjectivity are particularly challenging for the trainee sonographer, who is concurrently trying to perform the psycho-motor skill, operate the ultrasound machine and address technical or patient presentation difficulties.

With these challenges in mind, Sections 1a and 1b of the assessment and feedback package were developed to comprise the shoulder diagnostic ultrasound scan protocol (i.e. how to do the scan plus helpful tips) and the definition of scan findings and differential diagnosis (i.e. how to interpret the images plus helpful tips). The scan protocol (Section 1a) is based on the ESSR technical guidelines^10^ and adapted as per the clinical practice of the authors. The definition of scan findings and differential diagnosis (Section 1b) details how the expert interprets the scan images and arrives at a differential diagnosis. This was based on accepted sonographic interpretation of images and the formulation process of a differential diagnosis employed by the authors.

In this regard, the characterising of rotator cuff tears was based upon the work of McNally^12^ and defined according to the thickness, width and location of the tear. Tear thickness was described as partial (does not extend through full tendon thickness) and full (does extend through full tendon thickness) where tear thickness was defined for the supraspinatus tendon as communication between the joint and bursal surfaces. Location of the tear was defined relative to the portions of the tendon involved (e.g. subscapularis = superior, middle and inferior; supraspinatus = anterior/middle/posterior; bursal/intra-substance/articular side). Via longitudinal and transverse images of the relevant tendon, the width (and depth) of the tear was estimated via calliper measurements. Complete tears were defined by involvement of the full width of the whole of the tendon ± retraction. Furthermore, the condition of (any) remaining tendon was recorded via the prompt to identify any tendinopathic change and the presence of muscle belly atrophy was also prompted for.

It is acknowledged within the medical literature that whilst experts have tacit knowledge, it can be difficult for the learner to elicit that knowledge.^13^ Section 1b therefore supports the trainee by distilling down the tacit clinical reasoning knowledge of the trainer.

In developing the material, the authors were mindful that certain aspects of the scan are technically challenging (e.g. dynamic imaging of the lateral aspect of the cuff during humeral abduction; the subacromial impingement test), some require extensive scanning experience (e.g. judgements on tendon thinning/thickening), some provide confirmatory or secondary information (e.g. imaging the lateral aspect of the cuff to identify if the rotator cuff tissue is preserved) and some are areas of controversy within MSK sonography (e.g. tendinitis versus tendinosis and how these are defined and quantified). In order to support both the early stage and the more advanced trainee, the material was therefore subdivided to reflect this.

## Section 1c: Shoulder diagnostic ultrasound scan – Patient findings pro forma

The findings from a diagnostic ultrasound scan are typically collated in a written format by the sonographer for reporting back to the referring clinician. However, there can be great variability between MSK sonographers in the content and format of their reports and such inconsistency within sonography creates a potential barrier to effective communication. Furthermore, there is the highly contentious issue of sonographic findings versus clinical and symptomatic interpretation. For the trainee, it is important to quantify the accuracy of their scan findings compared to their trainer.

To provide consistency for the trainee focusing solely on sonographic findings and to facilitate quantification of accuracy, a pro forma was developed that reflects the terminology and format of the scan protocol (Section 1a) and definition of scan findings and differential diagnosis (Section 1b). This was adapted from a pre-existing pro forma that had been developed and successfully used for the training of qualified sonographers new to MSK ultrasound. The first pro forma facilitated assessment of diagnostic competence against specific structures in the shoulder. This allowed kappa values to be calculated quantifying student expertise in absolute terms against an expert practitioner.^14^ The new pro forma was expanded to include the assessment of technical competence for students new to the discipline of ultrasound. Explicitly linking how the scan is performed and interpreted, with how it is recorded and assessed, is an example of constructive alignment, an approach widely advocated within the medical education literature.^15^ Furthermore, the use of terminology linked to interpretive criteria (Section 1b) provides a degree of consistency when communicating sonographic findings. Two copies of Form 1c are therefore used for each patient (one completed by the trainee and one by the trainer), thereby allowing for direct comparison of findings and interpretation.

## Sections 2a and 2b: Assessment form for performance of shoulder diagnostic ultrasound scan, with assessment criteria

In clinical practice, the difficulty of performing a diagnostic ultrasound scan can be influenced by factors such as the ability of the patient to achieve and maintain optimum imaging positions. Subcutaneous fat levels and how well the patient’s tissues image along with the complexity of the pathological presentation and whether normal architecture has been preserved can also be a factor.^1,6^ These are all part of and influence the learning curve of the trainee sonographer. To reflect this, the first part of Section 2 involves the trainer rating the difficulty and complexity of the scan. In so doing, the package acknowledges the natural variation in patient presentations and the challenges these bring to learning an advanced clinical skill.

Identifying when a trainee sonographer is competent is a critical aspect of any ultrasound course and has obvious implications for patient care.^3^ Outside of a formal postgraduate course framework, clinicians such as orthopaedic surgeons evidence their competency using a binary yes/no measure of their accuracy in diagnosing rotator cuff tears compared to arthroscopic data.^16,17^ However, competency is a much more complex aspect of sonographic performance and includes technical performance of the skill, tissue and pathology identification and differential diagnosis.^18^ Furthermore, the diagnostic expertise of a trainee sonographer requires diverse pathologies to be recognised such as bursitis and tendinopathy, each of which requires specific technical skills to elicit and experience to interpret.

Competency of a level 1 sonographer is typically determined by using a log book (emphasising the breadth of experience acquired and number of scans performed) and examination.^18^ However, to the best of the authors’ knowledge, no criteria have been developed to quantify or measure such competency in MSK sonography. To address this, Section 2 therefore involves the trainer rating the technique, tissue and pathology identification performance of the trainee on the continuum from novice to competent ultrasound practitioner. Criteria for this judgement were adapted from pre-existing descriptors used in the assessment of abdominal and obstetric & gynaecological ultrasound trainees in a CASE approved postgraduate diagnostic ultrasound course.^19^

Whilst the trainee is performing their scan, the trainer therefore completes Form 2a. Assessing the technical competency of the trainee, whilst taking into account the difficulty and complexity of the scan, therefore ensures that the performance of the trainee (and their accuracy, as determined by the use of Form 1c) is continually and comprehensively assessed against a spectrum of patient presentations.

## Section 3: Combined performance assessment and scan findings form – For each tissue

As with other MSK regions, the imaging of each tissue in the shoulder has its own technical and pathological challenges for the trainee sonographer. For example, the acromio-clavicular joint can be readily palpated is imaged with the patient’s arm at rest and requires minimal probe manipulation to achieve optimal images. Conversely, the differentiation of healthy tendon from tendinopathic and subtle, partial thickness tears of the supraspinatus requires careful probe manipulation, management of anisotropy, optimal patient positioning (or adaptation if the patient cannot achieve this) and a well-reasoned differential diagnosis process.

For the more advanced trainee, Section 3 therefore enables the trainer to provide more specific feedback for each of the tissue regions being imaged. For each patient, a copy of Form 1c is completed by the trainee whilst a copy of Form 3a is completed by the trainer. The competency ratings make use of an amalgamated version of the assessment of trainee technique criteria, i.e. a combination of rating the technique and also tissue and pathology identification performance. Alongside this, the trainer also rates the difficulty and complexity of the scan. Over time therefore, the trainer would expect the trainee to move from novice to competent ratings across a spectrum of scan complexities. Where residual areas of difficulty persist (e.g. consistently low ratings of competency for the imaging of a particular tissue or patient type), the assessment form supports the trainee and trainer in identifying these as a focus for more targeted training support.

## Applications, limitations and recommendations

The training, assessment and feedback package detailed in this article has been developed using medical education principles to facilitate the effective training of professionals who seek to learn diagnostic MSK ultrasound of the shoulder. They provide a mechanism for trainees to follow an internationally recognised scanning protocol with supplementary information, which provides a logical framework for the formulation of a differential diagnosis.

The package supports the trainee with recording their findings in a non-onerous and consistent manner. It also helps the trainee and trainer to acknowledge the challenges of scanning different patients and pathological presentations. It provides a mechanism for trainers to quantify and trainees to evidence their emerging competency. The package also supports trainees and trainers to work collaboratively to identify problem areas for additional focus. The package detailed in this article is therefore proposed for use in shoulder ultrasound training and its principles could be adapted for other MSK regions or other ultrasound disciplines.

Limitations of the package include heavy reliance on the trainer to provide consistent judgements of technique, report accuracy and scan complexity. Successful use of the package is also dependent upon a significant time investment of an appropriately qualified and experienced trainer throughout the training period. This is a long-standing challenge within ultrasound training.

The diagnostic criteria used (particularly in Section 1c) are often binary choices, e.g. tendinopathic change yes/no. Whilst this may oversimplify the nature of ultrasound diagnosis, the pro forma does provide an opportunity for recording more subjective impressions. Nonetheless, establishing greater consensus in MSK ultrasound diagnosis is an important area for future work. It is also acknowledged that the pro forma relates only to the sonographic findings and not to clinical interpretation. Generating a technical sonographic report means that the trainee is only, therefore, required to focus on the images, without being drawn into controversies that surround the clinical significance of many sonographic findings.^20–22^

Utility of the assessment criteria (both the difficulty and complexity of the scan and the performance of the ultrasound practitioner) are currently being tested and preliminary results have been presented which demonstrate the pro formas’ efficacy.^14^ Formal reliability testing of the differential diagnosis process, grading of scan complexity and trainee performance are areas for future work. However, the reproducibility of the criteria will inevitably be influenced by the trainer and their training background. Nonetheless, a pre-defined, unified approach to training within and amongst different professions will not only improve this but also ensure greater homogeneity of clinical utility of the modality.

Ideally, the package should be used within a CASE approved post-graduate diagnostic ultrasound course framework as this ensures that the trainee receives broad-based and thorough training in the modality. However, this may not be feasible for all aspirant trainees. The package could therefore be utilised in a less formal training environment and provide evidence for professional registration, indemnity insurance and career development. However, the authors strongly advocate that trainees train under formally qualified sonographers, that trainees work within their scope of practice and that trainees maintain close links with radiology departments and formally qualified sonographers throughout their clinical practice as a way of benchmarking performance.

## Declarations

**Competing interests:** The authors have no conflicts of interest to declare.

**Funding:** This work was supported by Arthritis Research UK, Grant Number 19876.

**Ethical approval:** Not applicable.

**Guarantor:** MS.

**Contributorship:** MS conceived the paper and designed the education and assessment components. AR, NA, JK developed the educational assessment material, which was adapted for the paper. AH provided expert opinion on the musculoskeletal sonography content. PM provided expert opinion on the musculoskeletal sonography content and was the consultant radiologist with whom the training was undertaken. PM also developed the initial reporting form. MS wrote the manuscript. All authors reviewed and approved the final version.

## Appendix. A training, assessment and feedback package for the trainee shoulder sonographer

### Section 1a: Shoulder diagnostic ultrasound scan protocol (i.e. how to do the scan + helpful tips)

**Table:**

Step *(ESSR)*	Image/tissue	Additional information on how to do the scan (*+ helpful tips*)	Area of controversy or advanced/confirmatory technique
1*(2)*	Transverse view of biceps tendon	Identify tendon in inter-tubercular groove (*confirm target tissue by demonstrating anisotropy*). Follow distally to myo-tendinous junction and superiorly to GHJ. Is the tendon present/torn/displaced?	Is there evidence of tendinopathic change or tenosynovitis?
2*(2)*	Longitudinal view of biceps tendon	Identify tendon (*confirm target tissue by locating it between the longitudinal view of the greater and lesser tuberosities’ superficial cortical bone margins*). Ensure tendon fibres are parallel to screen image by “heeling in” the probe distally. Follow distally to myo-tendinous junction and superiorly to GHJ. Is the tendon present/torn/displaced?	Is there evidence of tendinopathic change or tenosynovitis?
3	Dynamic transverse assessment of biceps tendon	Tendon – Is it stable in inter-tubercular groove or displaced/subluxed, during GHJ internal/external rotation? (*If patient has limited GHJ external rotation then ask them to perform GHJ internal rotation*)	
4*(3)*	Longitudinal view of Subscapularis	Identify tendon (*use shoulder extension if required, to visualise superior border*). Sweep through superior, middle and inferior portions; tendinous insertion through to myo-tendinous junction (if possible). Is the tendon present/torn (partial/full thickness/complete; particularly superior third)?	Is there evidence of tendinopathic change, including calcific deposits? Estimate dimensions of tear via callipers.
5*(3)*	Transverse view of Subscapularis	Identify tendon (*use shoulder extension if required, to visualise superior border*). Sweep from insertion through to myo-tendinous junction (*use LHB to ensure superior edge of subscapularis tendon has been imaged*). Is the tendon present/torn (partial/full thickness/complete; particularly superior third)?	Is there evidence of tendinopathic change, including calcific deposits? Estimate dimensions of tear via callipers.
6*(10)*	Acromioclavicular joint	Identify joint. Is it osteophytic/irregular; is capsular hypertrophy present; localised vascular signal present?	Is there local tenderness upon scanning?
7*(5)*	Transverse view of Supraspinatus	Identify LHB in transverse section – Denotes rotator interval (*from which the anterior edge of supraspinatus can be identified*). Sweep the probe from antero-medial to postero-lateral to scan through the ant/mid/post-fibres. Sweep inferiorly to insertion and superiorly towards muscle belly. Is tendon present; is there a tear (partial/full thickness/complete)? For tears – location: ant/mid/post; bursal/intra-substance/articular side Mark on diagram with X.	Is there evidence of tendinopathic change, including calcific deposits? Mark on diagram with O Bursa – is there debris or increased fluid/thickening? Estimate dimensions of tear via callipers.
8*(5)*	Longitudinal view of Supraspinatus	Identify LHB in oblique section (*use absence of greater tuberosity to differentiate LHB in oblique section* from *Supraspinatus fibres; LHB has a more fibrillar pattern*). Scan insertion to muscle belly; sweep to ant/mid/post–fibres If tendon is present, is there evidence of tear (partial/full thickness/complete)? Location: ant/mid/post; bursal/intra-substance/articular side.	Is there evidence of tendinopathic change, including calcific deposits? Bursa – Is there debris or increased fluid/thickening? Estimate dimensions of tear via callipers.
11*(7)*	Subacromial impingement test + visualisation of tissue in subacromial region		Identify subacromial tissue. Fix probe in the coronal plane and observe motion during active abduction of the humerus. With humerus rested by side, image ant/mid/post–supra and through to infra. Is acromio-humeral distance/presence of rotator cuff tissue preserved in the observable region?
9*(8)*	Transverse view of Infraspinatus (and Teres Minor)	Identify muscle through to tendon – sweep medially/laterally for diverging/converging muscle bellies (respectively). Tendon tear (partial/full thickness/complete)?	Observe muscle thickness and fatty infiltration Is there evidence of tendinopathic change, including calcific deposits? Estimate dimensions of tear via callipers.
10*(9)*	Longitudinal view of Infraspinatus (and Teres Minor) Posterior glenohumeral joint recess	Identify tendon – Sweep laterally to identify insertion and also posterior glenoid Tear (partial/full thickness/complete)? Joint effusion or labral cyst?	Is there evidence of tendinopathic change, including calcific deposits? Estimate dimensions of tear via callipers

Note: *EESR* indicates the corresponding step in the ESSR guidelines; GHJ: glenohumeral joint; LHB: long head of biceps. Step number 11 is included as an advanced/confirmatory technique only. However it is placed immediately after step 8 because it relates particularly to exploration of tissues imaged in that step.

### Section 1b: Definition of scan findings and differential diagnosis (i.e. how to interpret the images + helpful tips)

**Table:**

Step *(ESSR)*	Image/tissue	Definition of findings/differential diagnosis (*+ helpful tips*)	Area of controversy or advanced/confirmatory technique
1*(2)*	Transverse view of biceps tendon	• Tendon present and normal – Visible as hyper-echoic structure; characterised by anisotropy. • Tendon torn (partial) – Hyper-echoic structure located but abrupt change in cross-sectional shape or size; location of change reported in relation to bicipital groove. • Tendon torn (complete) – Hyper-echoic structure cannot be located (*requires confirmation in two planes*). • Tendon displaced – Present, but not in inter-tubercular groove; dislocates.	• Tendinopathic change – Thickening or thinning of tendon; hypo-echoic, irregular signal/fibre appearance. • Tenosynovitis –thickening of synovial sheath and/or increased fluid.
2*(2)*	Longitudinal view of biceps tendon	• As per step 1.	• As per step 1.
3	Dynamic transverse assessment of biceps tendon	• Tendon stable in inter-tubercular groove. • Tendon displaced/subluxes, during GHJ internal/external rotation.	
4*(3)*	Longitudinal view of Subscapularis	• Tendon present and normal – Visible by characteristic shape and attachment to lesser tuberosity. • Tendon torn (partial) – Hypo-echoic region which does not extend through full tendon thickness (confirm in two planes), +/− fluid or tissue in-fill. • Tendon torn (full) – Hypo-echoic region which does extend through full tendon thickness (confirm in two planes), +/− fluid or tissue in-fill. • Tendon torn (complete) – Hypo-echoic region which extends through full tendon thickness and width +/− retraction (confirm in two planes), +/− fluid or tissue in-fill.	• Tendinopathic change – Thickening or thinning of tendon; hypo-echoic, irregular signal/fibre appearance; calcific deposits.
5*(3)*	Transverse view of Subscapularis	• Tendon present and normal – Visible by characteristic shape and fibrillar arrangement (*note fibrillar arrangement is a normal finding and should not be confused with tendinopathic change or tear*). • Tendon torn (partial, full or complete) – As per step 4 (above); confirm tear in two planes.	• Tendinopathic change – Thickening or thinning of tendon; hypo-echoic, irregular signal/fibre appearance; calcific deposits.
6*(10)*	Acromioclavicular joint	• Joint margins smooth and normal. • Osteophytic – Irregular joint margins with bony outgrowths; graded according to size and irregularity of outgrowths. • Synovitis/Inflammation – Capsular hypertrophy; localised vascular signal present (*independent of patient breathing/talking*).	• Does the patient report reproduction of their symptoms upon scanning or pressure from probe?
7*(5)*	Transverse view of Supraspinatus	• Tendon present and normal – Visible by characteristic appearance: • Tendon torn (partial) – Hypo-echoic region which does not extend through full tendon thickness (confirm in two planes), +/− fluid or tissue in-fill; identify bursal/intra-substance/articular side. • Tendon torn (full) – Hypo-echoic region which does extend through full tendon thickness (confirm in two planes), +/− fluid or tissue in-fill; identify bursal/intra-substance/articular side. • Tendon torn (complete) – Hypo-echoic region which extends through full tendon thickness and width +/− retraction (confirm in two planes), +/− fluid or tissue in-fill.	• Tendinopathic change – Thickening or thinning of tendon; hypo-echoic, irregular signal/fibre appearance; calcific deposits. • Bursa – Fluid present (hypo-echoic)/thickening; debris present (mixed echogenicity).
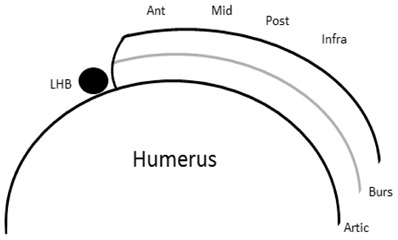
8*(5)*	Longitudinal view of Supraspinatus	• Tendon present and normal – Visible by characteristic “birds beak” shape and attachment to greater tuberosity. • Tendon torn (partial/full thickness/complete) – As per step 7; confirm in two planes.	• Tendinopathic change – thickening or thinning of tendon; hypo-echoic, irregular signal/fibre appearance; calcific deposits. • Bursa – Fluid present (hypo-echoic)/thickening; debris present (mixed echogenicity).
11*(7)*	Subacromial impingement test + visualisation of tissue in subacromial region		• Is there smooth passage of subacromial tissues under the acromion? • Does the patient report reproduction of their symptoms when moving? • From anterior to posterior subacromial region, is acromio-humeral distance preserved/tendon tissue present throughout the observable region?
9*(8)*	Transverse view of Infraspinatus (and Teres Minor)	• Muscle(s) through to tendon(s) present and normal (*visible by characteristic shape*). • Tendon torn (partial) – Hypo-echoic region which does not extend through full tendon thickness (confirm in two planes), +/− fluid or tissue in-fill. • Tendon torn (full) – Hypo-echoic region which does extend through full tendon thickness (confirm in two planes), +/− fluid or tissue in-fill. • Tendon torn (complete) – Hypo-echoic region which extends through full tendon thickness and width +/− retraction (confirm in two planes), +/− fluid or tissue in-fill.	• Estimate if muscle thickness is within normal limits; loss of marbled muscle appearance for fatty infiltration. • Tendinopathic change – Thickening or thinning of tendon; hypo-echoic, irregular signal/fibre appearance; calcific deposits.
10 *(9)*	Longitudinal view of Infraspinatus (and Teres Minor) Posterior glenohumeral joint recess	• Tendon(s) present and normal – Visible by characteristic shape and attachment to greater tuberosity; identify posterior glenoid. • Tear (partial/full/complete)? • Joint effusion or labral cyst?	• Tendinopathic change – Thickening or thinning of tendon; hypo-echoic, irregular signal/fibre appearance; calcific deposits.

Note: *EESR* indicates the corresponding step in the ESSR guidelines. Where a structure (or “change” in a structure) has been identified as a pathological finding and/or potential cause of pain, then comparison with the structure of interest on the contralateral side should be performed. It is acknowledged that asymmetry can be a “normal” finding; nonetheless, comparison with the contralateral side can provide useful confirmatory information.
